# Debt and Death

**Published:** 1855-03

**Authors:** 


### DEBT AND DEATH.

General Jackson once said that any man who traded on a borrowed capital ought to break; be that as it may, I consider him a radically dishonest man who embarks in business wholly on a borrowed capital, because he is willing to endanger his friend for the chance of his own profit; he cannot lose, but his friend may. James Harper, one of the best and purest men I ever knew, a Virginia gentleman, of the old school, whose heart was welling up unceasingly with human kindness to all around him, once said to a gentleman who counted his fortune by hundreds of thousands, and who voluntarily offered to be drawn upon for any amount, replied: "*I can't consent to make a fortune at the risk of my friend.*" A mercantile gentleman, who was honor personified, *Joseph Stephens*, once said to me: "I leave my bed of a morning bathed in perspiration, in the agony of device for meeting the engagements of the day." We all know that the fear of not being able to meet pecuniary engagements is a frequent cause of insanity and suicide to men of refinement and of a high sense of honor, while thousands are wasting away around us under the harassing pressure of debt. The temper is uneven; at one time sad, at another almost unendurably irritable; the appetite is variable, if any at all; the nights are restless, the sleep unrefreshing; gladness hies from home, and silent gloom pervades the fireside circle, thus verifying the scripture assertion that *they who hasten to be rich shall pierce themselves through with many sorrows.*

In view then of its health-destroying influences, I may very properly give the admonition in this journal, *avoid debt:* shun it as you would the pestilence that walketh in darkness, or the plague that wasteth at noonday; consider it your mortal enemy—the enemy of your body, your health, your happiness, your soul—the enemy of your wife, your children, and every kindred tie.

Take almost any business man, and he will tell you in more than three cases out of four, that he has lost more by bad debts than he is now worth. It is a monstrous fallacy that "if a man expects to become rich he must go in debt." The sentiment originated in the heart of a rogue. Debt is not the policy of the most successful men.

I adopt, with all my heart, a paraphrase of a favorite expression of President Lindsley, which I have treasured in my own mind for more than a quarter of a century, "I dictate to no man, and allow no man to dictate to me." I go in debt to no man, I allow no man to go in debt to me. Who can for a moment doubt that if this were the prevalent sentiment and practice of the time, half of all the sorrow that now palls humanity's heart, would be instantaneously annihilated. Men would not get on quite so fast in their improvements, in building palatial residences, and opening splendid farms, but they would get along more surely, and in the end lie down to die with a happier heart by far, and have no quenchless remorses to embitter the last moments of life. Mr. Everett states in his memoir of Peter C. Brooks, of Boston, who died worth millions, "that Mr. B. abstained as a general rule, from speculative investments; 'his maxim was, that the whole value of wealth consisted in the personal independence which it secured, and he was never inclined to put that good, once won, again at hazard, in the mere quest of extraordinary additions to his superfluity.' He never made purchase of unproductive real estate, on a calculation of future enhanced value. He never directly or indirectly took more than legal interest. He could have doubled his immense fortune had he been willing to violate this rule. It is mentioned that he believed and often said, that, 'in the long run,' six per cent is as much as the bare use of money is worth in this country. It was another of his principles never himself, to borrow money. What he could not compass by present means was to him interdicted. It is doubtful whether, with but a single exception, Mr. Brooks' name was ever subscribed to a note of hand. He shunned every transaction, however brilliant the promise of future gain, which required the use of borrowed means. Mr. Everett well remarks:

"The bold spirit of modern enterprise will deride as narrow minded so cautious a maxim; but the vast number of individuals and families actually ruined by its non-observance—to say nothing of the heaven-daring immoralities so often brought to light, to which men are tempted in the too great haste to be rich—go far to justify Mr. Brooks' course. It is highly probable, that, in the aggregate, as much property is lost and sacrificed in the United States by the abuse of credit, as is gained by its legitimate use. With respect to the moral mischiefs resulting from some of the prevailing habits of our business community—the racking cares and corroding uncertain ties, the mean deceptions, and the measureless frauds to which they sometimes lead—language is inadequate to do justice to the notorious and appalling truth."

With all his rare excellencies of christian character, there were few men wiser in this world's wisdom than the late Rev. Dr. Milner. His long practice at the bar, and his experience as a politician, in and out of Congress, peculiarly qualified him to judge of human nature and of the tendency of things, and to give prudent advice. "My next door neighbor is in debt. Upwards of two years ago he borrowed from me two hundred dollars, and immediately afterwards one hundred and ten more. The latter sum he engaged to return in twenty-four hours. I have never received a shilling of those sums in money; but as he is a bookseller, I have, at his urgent solicitation, taken books of him to the amount of nearly two-thirds of the demand. His note for the balance is now due, and he urges me to take Viner's Abridgment, which satisfies the debt, except thirty or forty dollars.

"During the whole time since the loan, he has persevered in a system of cringing prevarication and promises, which he must have known at the time he dealt them out, he never would fulfil. Various artifices, false tales, shifts, and pretences he has made use of; and I have been the dupe of them. I cannot believe him to be so destitute of feeling as not to be mortified and degraded in his own estimation, by the imagined necessity of resorting to them. But in the one case or the other, I am unable to point to myself a more humiliating situation for a human being to stand in.

"I have derived from this transaction two pieces of instruction, which are, in my view, an adequate compensation for the whole sum, had such an event happened:—

1. To be cautious of hastily and unadvisedly lending money to a man of whose ability and punctuality I am not well assured, unless it be accompanied by adequate security.

2. To adhere religiously to a determination which I formed at the moment of commencing business, never to incur debt which I have the remotest apprehension of being unable, or even finding it inconvenient to discharge. And, in order constantly to possess the means of keeping this resolution, whatever my income may be, always to live within it."

